# Draft Genome Sequence of *Sphingopyxis* sp. Strain MWB1, a Crude-Oil-Degrading Marine Bacterium

**DOI:** 10.1128/genomeA.01256-14

**Published:** 2014-12-04

**Authors:** Jonghyun Kim, Soo Jung Kim, Seon Hee Kim, Seung Il Kim, Yoon-Jung Moon, Sung-Joon Park, Hyung-Yeel Kahng, Young-Ho Chung

**Affiliations:** a Division of Life Science, Korea Basic Science Institute (KBSI), Daejeon, South Korea; b Department of Environmental Education, Sunchon National University, Suncheon, South Korea

## Abstract

*Sphingopyxis* sp. strain MWB1, which is capable of degrading crude oil, diesel, and kerosene, was isolated from crude oil–contaminated seashore in Tae-an, South Korea. Here, we report the draft genome sequence of this strain, which comprises 3,118,428 bp with a G+C content of 62.85 mol%.

## GENOME ANNOUNCEMENT

The genus *Sphingopyxis* belongs to the group sphingomonads. Sphingomonad strains are strict aerobic, rod-shaped, chemoheterotrophic, Gram-negative, and typically yellow-pigmented bacteria, and they are able to degrade various xenobiotic substrates. The genus *Sphingomonas* has been classified into at least four clusters within the α-4 subclass of the *Proteobacteria* and has also been divided into four genera—*Sphingomonas*, *Sphingobium*, *Novosphingobium*, and *Sphingopyxis*—based on phylogenetic, chemotaxonomic, and phenotypic differences (1, 2). The genus *Sphingopyxis*, which is currently classified by 20 species (http://www.straininfo.net), was isolated from a variety of environments, including seawater, a hexachlorocyclohexane-contaminated dumpsite, landfill soil, wastewater, and so on (3–6). This genus has been known to be able to degrade a broad range of mono- and polycyclic aromatic hydrocarbon compounds (7–9).

Recent severe oil spills in the marine environment have given us the opportunity to isolate indigenous oil-degrading microorganisms and apply them to the bioremediation of oil contaminants. Consequently, the strain *Sphingopyxis* sp. MWB1—isolated from the seashore in Tae-an, South Korea, that was contaminated by a crude-oil spill—was able to degrade crude oil, diesel, and kerosene. Little has been known about the genes and enzymes responsible for the degradation of petroleum derivatives in spite of its capabilities, which prompted us to determine the genome sequence of the strain MWB1.

The genome of the *Sphingopyxis* sp. MWB1 strain was sequenced using the MiSeq 250 bp paired-end 250 platform (Illumina Co., United States) at ChunLab, Inc. (Seoul, South Korea). A total of 15,202,637 reads were generated from the Illumina system, resulting in 1,046.38 sequencing coverage. The sequence data were assigned to five contigs by CLCbio Genomics Workbench version 6.0 (CLCbio, Denmark), and a 3,118,428 bp draft sequence was established with an *N*_50_ contig length of 2,542,167 bp and a G+C content of 62.85 mol%. The draft genome sequence was annotated by Glimmer 3, HMM search 2, and tRNA-scan for CDS, rRNA, and tRNA prediction, and by the rpsBLAST program using the NCBI COG database (http://www.ncbi.nlm.nih.gov/COG) and the Rapid Annotation using Subsystem Technology (RAST) server with SEED database for gene annotation (10, 11). As a result, the genome of this strain codes 2,965 CDSs, 1 rRNA, and 45 tRNAs. In addition, the *Sphingopyxis* sp. MWB1 strain shared a 98.39% similarity with *Sphingopyxis witflariensis* W-50(T), based on the 16S rRNA gene sequence.

### Nucleotide sequence accession numbers.

The draft genome sequence of *Sphingopyxis* sp. MWB1 has been deposited at DDBJ/EMBL/GenBank under the accession number JQFJ00000000. The version described in this paper is version JQFJ01000000.
